# Editing the Shape Morphing of Monocomponent Natural Polysaccharide Hydrogel Films

**DOI:** 10.34133/2021/9786128

**Published:** 2021-06-02

**Authors:** Hao Hu, Chao Huang, Massimiliano Galluzzi, Qiang Ye, Rui Xiao, Xuefeng Yu, Xuemin Du

**Affiliations:** ^1^Institute of Biomedical & Health Engineering, Shenzhen Institute of Advanced Technology (SIAT), Chinese Academy of Sciences (CAS), Shenzhen 518035, China; ^2^Key Laboratory of Polymeric Materials and Application Technology of Hunan Province, Key Laboratory of Environmentally Friendly Chemistry and Applications of Ministry of Education, School of Chemistry, Xiangtan University, Xiangtan 411105, China; ^3^Institute of Advanced Materials Science and Engineering, Shenzhen Institute of Advanced Technology (SIAT), Chinese Academy of Sciences (CAS), Shenzhen 518035, China; ^4^State Key Laboratory of Fluid Power & Mechatronic System, Key Laboratory of Soft Machines and Smart Devices of Zhejiang Province, Department of Engineering Mechanics, Zhejiang University, Hangzhou 310027, China

## Abstract

Shape-morphing hydrogels can be widely used to develop artificial muscles, reconfigurable biodevices, and soft robotics. However, conventional approaches for developing shape-morphing hydrogels highly rely on composite materials or complex manufacturing techniques, which limit their practical applications. Herein, we develop an unprecedented strategy to edit the shape morphing of monocomponent natural polysaccharide hydrogel films via integrating gradient cross-linking density and geometry effect. Owing to the synergistic effect, the shape morphing of chitosan (CS) hydrogel films with gradient cross-linking density can be facilely edited by changing their geometries (length-to-width ratios or thicknesses). Therefore, helix, short-side rolling, and long-side rolling can be easily customized. Furthermore, various complex artificial 3D deformations such as artificial claw, horn, and flower can also be obtained by combining various flat CS hydrogel films with different geometries into one system, which can further demonstrate various shape transformations as triggered by pH. This work offers a simple strategy to construct a monocomponent hydrogel with geometry-directing programmable deformations, which provides universal insights into the design of shape-morphing polymers and will promote their applications in biodevices and soft robotics.

## 1. Introduction

In nature, shape-morphing behaviors of organisms are vital for their survival [1]. Typically, pinecones and peasecod can adjust their morphologies in response to humidity for releasing their seeds at appropriate periods [2]. This intriguing shape morphing results from the mismatch strain/stresses in local swelling behaviors which are determined by anisotropic tissue compositions and micro-/nanostructures within the organisms [3–5]. Inspired by these smart behaviors in nature, efforts have been focused on the development of artificial materials with programmable shape-morphing capabilities [6], such as electroactive polymers [7], liquid crystal elastomers [8], shape memory polymers [9], and stimuli-responsive hydrogels [10], which hold great promises to develop artificial muscles [11], reconfigurable tissue engineering scaffolds [12–14], and soft robotics [15–17] for a broad range of applications. Among these, hydrogels are one of the most attractive candidates, because they not only can swell and shrink in response to certain stimuli (thus changing their volumes reversibly) but also can exhibit unique superiorities in high similarity (water-rich structures and mechanical properties) to soft biological tissues, making hydrogels the most promising candidates in biomedical and robotic fields [18–20].

In recent years, shape-morphing hydrogels have gained extensive attention [20, 21]. Conventionally, the shape-morphing hydrogels are realized by introducing diverse components with different swelling/shrinking behaviors upon exposure to specific stimuli. For example, bilayer hydrogels consisting of active and passive components are usually used to realize deformations based on the mismatch strain triggered by the swelling/shrinking of the active layer [22, 23]. However, the introduction of diverse components may suffer from intrinsic limitations, including unstable interfaces, time-consuming fabrication, and limited opportunities for biomedical applications [24, 25]. Therefore, it is greatly significant to prepare monocomponent hydrogel films enabling precisely controlled deformations. Typically, anisotropic structures and cross-linking densities either in plane or out of plane (across the thickness) are rationally designed into monocomponent hydrogel films to induce programmable shape morphing [26, 27]. Nevertheless, the complex anisotropic designs are highly dependent on various advanced manufacturing techniques, such as photomask-assisted photopolymerization [28], and 3D/4D printing [29–31], to tune the mismatch strain/stresses, resulting in high cost and low versatility. In view of the intrinsic influence of the geometry effect (i.e., length-to-width ratio and thickness) on the mismatch strain/stresses [32–34], we therefore hypothesize that the shape morphing of monocomponent natural polysaccharide hydrogel films could be edited solely by modulating their geometries.

To confirm this hypothesis, we develop an unprecedented strategy to integrate gradient cross-linking density and geometry effect into a monocomponent natural polysaccharide hydrogel film, chitosan (CS), which has remarkable advantages of excellent biocompatibility and functionalities [35, 36]. Upon swelling or shrinking of CS hydrogel films, the gradient cross-linking density across the thickness results in different swelling/shrinking ratios, which further leads to the mismatch strain/stress. The out-of-plane strain/stress finally induces bending deformations of CS hydrogel films, which can be modulated via the geometry effect, leading to controllable three-dimensional (3D) deformations in water or solutions with various pH values. Due to the synergistic effect, the shape morphing of CS hydrogels can be facilely edited by changing their geometries (length-to-width ratios or thicknesses). Therefore, helix, short-side rolling, and long-side rolling can be easily customized. Furthermore, various complex artificial 3D deformations such as artificial claw, horn, and flower can also be obtained by combining various planar CS hydrogel films with different geometries into one system, of which shapes can further be altered by pH. Our studies not only offer a simple yet effective strategy for precise controlling over the deformations of mono-component hydrogel films (e.g., CS and sodium alginate (SA) hydrogels) but also present universal insights into the geometry-directing programmable deformations, which will provide valuable guidelines for the design and developments of shape-morphing polymers, soft actuators, and reconfigurable biodevices.

## 2. Results

### 2.1. Fabrication of CS Hydrogel Films with Gradient Cross-Linking Density

**Figure 1:**
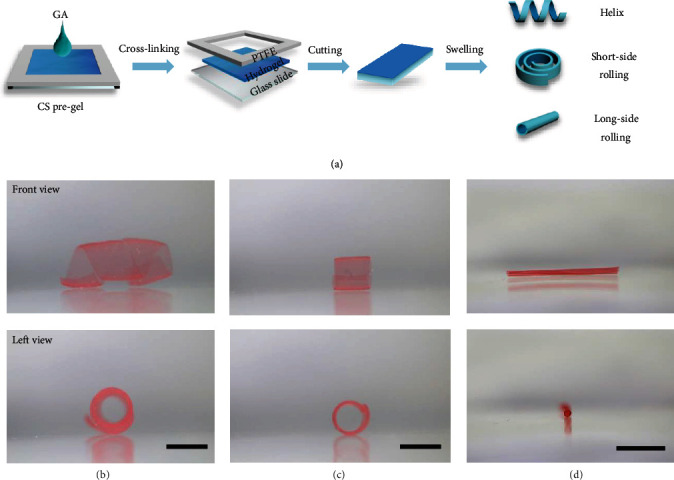
Fabrication and shape morphing of chitosan hydrogel films with gradient cross-linking density. (a) Schematic illustration of the preparation and various shape-morphing behaviors of chitosan hydrogel films with gradient cross-linking density. (b–d) show the front view and left view of various 3D programmable deformations of CS hydrogel films in deionized water with different geometries, including helix (3 mm × 24 mm × 80 *μ*m) (b), short-side rolling (3 mm × 9 mm × 60 *μ*m) (c), and long-side rolling (3 mm × 12 mm × 30 *μ*m) (d), respectively. The scale bars are 5 mm.

CS hydrogel films with gradient cross-linking density across the thickness are fabricated by top-down diffusion of glutaraldehyde (GA) molecules within a CS pregel solution. In a typical experiment (Figure 1(a)), CS powders were firstly dissolved in an acetic acid solution (2.0 wt%) to prepare CS pregel solution (2.2 wt%), which was then poured into a home-made device, and GA solution was subsequently dripped onto the top of the pregel solution. Thus, CS hydrogel was cross-linked via Schiff-base reaction between the amino groups of CS and the aldehyde groups of GA [37, 38]. After being placed at room temperature for 24 hours, the dried CS hydrogel film was peeled off and then cut into specific sizes, which could quickly change their shapes from planar to 3D structures after immersing into water in seconds (Figures 1(b)–1(d) and Figure S1). The deformed shape and swelling ratio can be kept in deionized water stably after complete removal of the residual acetic acid by a 0.01 M sodium bicarbonate solution (Figure S2). The shape morphing of CS hydrogel film is induced by the synergistic effect of the gradient cross-linking density across the thickness and geometry effect.

**Figure 2:**
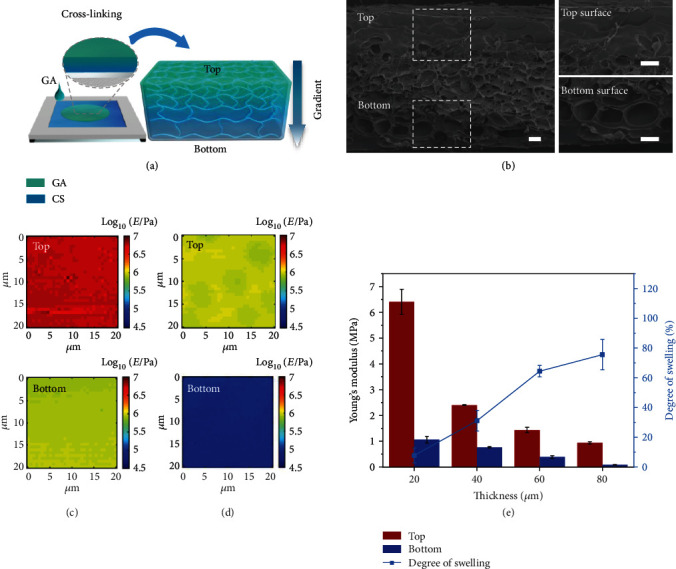
Characterization of CS hydrogel films. (a) The scheme shows the gradient cross-linking density across the thickness of CS hydrogel films. (b) The cross-sectional field-emission-scanning electron microscopy (FE-SEM) images of the frozen-dried CS hydrogel films and the related magnified images of the top and bottom surfaces (scale bar: 20 *μ*m). (c, d) Young's modulus mapping for the top and bottom surfaces of the swollen CS hydrogel films with different thicknesses (20 *μ*m and 80 *μ*m). (e) Young's modulus of both the top and bottom surfaces decreases as the hydrogel thicknesses increase (from 20 *μ*m, 40 *μ*m, 60 *μ*m, to 80 *μ*m). The degree of swelling also increases as the thickness of CS hydrogel films increases.

We first investigate the effect of the gradient cross-linking density across the thickness on the swelling property of CS hydrogel film. As shown in Figure 2(a), the amount of GA molecules diffused into CS hydrogel films determines the network density of CS chains. Consequently, CS chains form the tightest networks at the top surface owing to their complete exposure to the GA solution, leading to the highest cross-linking density. On the contrary, CS chains form looser networks at the bottom surface due to the decreasing GA molecules diffusible into the deeper sites of the hydrogel film, thus resulting in a lower cross-linking density and looser structure at the bottom surface than at the top surface (Figure 2(b)). Therefore, the gradient cross-linking density results from the GA concentration gradient across the thickness of the CS hydrogel film, which is further verified by the results of Young's modulus mapping. As shown in Figures 2(c)–2(e), Young's modulus at the top surface of the CS hydrogel film is always higher than that at the bottom surface, regardless of the changes in the thickness of the film (Table S1). Notably, Young's modulus of both the top and bottom surfaces decreases with increase of the thickness of the CS hydrogel film due to the increase of CS molecules involved in the cross-linking (Figure 2(e)). Specifically, for a thin hydrogel film, the GA molecules can reach the bottom surface of the hydrogel rapidly due to the short diffusion distance and thus have enough time to react with the CS hydrogel film on both sides. Consequently, the thin CS hydrogel film is saturated with the GA solution, resulting in high Young's modulus at both sides (for a 20 *μ*m thick hydrogel film, Young's modulus of the top surface: 6.41 ± 0.49 MPa, and Young's modulus of the bottom surface: 1.06 ± 0.13 MPa). In comparison, for a thick hydrogel film, the GA molecules need a long time to reach the bottom surface due to the long diffusion distance and thus have short time to react with the increased CS molecules across the thickness, resulting in a low Young's modulus at both sides (for an 80 *μ*m thick hydrogel film, Young's modulus of the top surface: 0.94 ± 0.05 MPa, and Young's modulus of the bottom surface: 0.08 ± 0.01 MPa). These distinct differences in stiffness between the top and bottom surfaces of the hydrogel films further confirm their different cross-linking densities across the thickness [39]. Moreover, these cross-linking density differences induce different swelling/shrinking ratios of CS hydrogel films (Figure 2(e) and Figure S2), which further leads to the mismatch strain/stress (out-of-plane strain/stress).

### 2.2. Mechanical Analysis of Shape-Morphing Hydrogels

**Figure 3:**
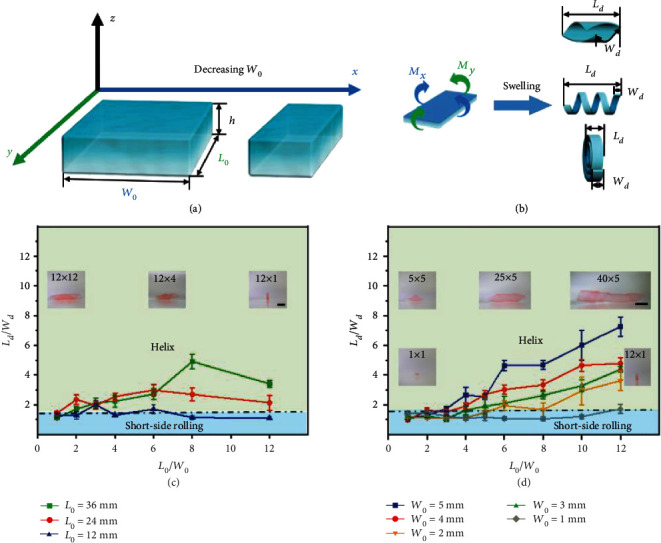
The shape-morphing behaviors of CS hydrogel films via changing the length-to-width (*L*_0_/*W*_0_) ratios. (a, b) Schematic illustration of the shape-morphing behaviors (bending moments of **M**_*x*_ at the *x*-axis direction, and **M**_*y*_ at the *y*-axis direction) of CS hydrogel films (certain thickness) via changing the length-to-width ratios. *L*_*d*_ and *W*_*d*_ are the length and width of the swollen CS hydrogel films, respectively. (c) The relationship between the *L*_0_/*W*_0_ and *L*_*d*_/*W*_*d*_ shows different shape-morphing behaviors of CS hydrogel films (as shown in the inserted images) with different *L*_0_ (36 mm, 24 mm, and 12 mm) in the same range of *L*_0_/*W*_0_ ratios. (d) The relationship between the *L*_0_/*W*_0_ and *L*_*d*_/*W*_*d*_ shows different shape-morphing behaviors of CS hydrogel films (as shown in the inserted images) with different *W*_0_ (1 mm, 2 mm, 3 mm, 4 mm, and 5 mm) in the same range of *L*_0_/*W*_0_ ratios. The scale bars are 5 mm.

As reported in our previous work [26, 28], solely out-of-plane strain/stress can lead to nonuniform bending of a planar hydrogel film upon swelling. It means that programming the bending directions of the hydrogel films into well-defined 3D shapes still requires precise tuning of the mismatch strain/stress at specific directions, which is highly relevant to not only the gradient cross-linking density across the thickness of the hydrogel films but also the original geometries (e.g., length-to-width ratio and thickness) of the hydrogel films [32]. To further understand the synergistic effect of the gradient cross-linking density and geometry effect on the shape-morphing behaviors, we analyze the bending moments induced by the internal stresses of the swelling or shrinking hydrogel films. The basic mechanics of the hydrogel film with gradient cross-linking density across the thickness and specific geometry (e.g., length-to-width ratio and thickness) can be simply described by the bimetallic strip model (Equation (1)) [40].

(1) $$\begin{array}{c} Μ=\frac{\text{EI}}{\rho }\propto k\Delta {lh}^{2}, \end{array}$$

where EI is the bending rigidity of a hydrogel layer, and *ρ* is the radius of the bending curvature. The **M** is positively related to the length difference before and after swelling of one side (Δ*l*) and square to the thickness of the hydrogel film (*h*) (Equation (1)), and *k* is a Young's modulus-related parameter. Unlike in a simple beam-like bilayer, the swelling of a hydrogel film with gradient cross-linking density across the thickness triggers a biaxial expansion inside the hydrogel, which creates mismatch strain/stress [32]. The resultant mismatch strain/stress further induces shape transformation of a hydrogel film, which is directed by the resultant bending moment (**M**_*xy*_) that includes **M**_*x*_ (**M**_*x*1_, **M**_*x*2_) along the *x*-axis and **M**_*y*_ (**M**_*y*1_, **M**_*y*2_) along the *y*-axis of the four sides of the hydrogel film (Figures 3(a) and 3(b)). By changing the geometry of a hydrogel film, e.g., initial length-to-width ratio (initial length/initial width: *L*_0_/*W*_0_) or thickness (*h*), the bending moments along the *x*-axis and/or *y*-axis will change due to the changing Δ*l* and *h* or the changing Young's modulus of the top and bottom surfaces of the hydrogel film, leading to the change of the resultant bending moment (**M**_*xy*_). Notably, the shape morphing of a hydrogel film is determined by the changed resultant bending moment, while the shape deformations always meet the request of lowest energy principle [34, 40–43].

### 2.3. Programmable Shape Morphing via Changing Length-to-Width Ratios

To reveal the geometry effect on the shape morphing of CS hydrogel films, we first investigate the influences of the length-to-width ratios (*L*_0_/*W*_0_) on the shape-morphing behaviors of CS hydrogel films with a certain thickness (80 *μ*m). As shown in Figure S3, three types of shape-morphing behaviors (diagonal rolling, helix, and short-side rolling) are observed. When *L*_0_/*W*_0_ is 1 (*L*_0_: 12 mm, 24 mm, and 36 mm), the hydrogel films prefer to demonstrate diagonal rolling (Movie S1). It results from the resultant bending moment (**M**_*xy*_) being along the diagonal direction, where the moment along the *x*-axis (**M**_*x*_) is close to the moment along the *y*-axis (**M**_*y*_). When the *L*_0_/*W*_0_ ratio increases from 2 to 12 via decreasing the initial width (*W*_0_) of CS hydrogel films, the hydrogel films mainly demonstrate helix structures, which have the lowest energy. Specifically, as the *L*_0_/*W*_0_ ratio increases slightly (<6), the diffusion of water inside the hydrogel layer is similar along both the length (*L*_0_) and width (*W*_0_) directions, resulting in bending simultaneously at these two sides. Afterwards, the bending along the length direction (*L*_0_) eventually dominated due to the slightly larger moment along this direction (**M**_*y*_), in agreement with energetic considerations, leading to a final helix shape. Comparatively, as the *L*_0_/*W*_0_ ratio increases largely (≥6), the diffusion of water inside the hydrogel layer is faster along the width direction (*W*_0_) than the length direction (*L*_0_), resulting in bending along the width direction (*W*_0_) first and then along the length direction. The bending (**M**_*x*_) along the width direction (*W*_0_) firstly occurs due to the fast swelling along this direction, and the bending (**M**_*y*_) along the length direction (*L*_0_) starts later due to the edge effects [34]. Thus, the collaborative bending (**M**_*xy*_) along these two directions leads to helix structures (Movie S2 and Movie S3). Notably, the edge effects become stronger as the *L*_0_/*W*_0_ increases largely, which would change the shape-morphing path. As shown in Figure S3, the hydrogel films (*L*_0_: 12 mm, *L*_0_/*W*_0_ ≥ 8) show short-side rolling as the *W*_0_ decreases to ~1 mm, which results from the much faster diffusion of water inside the hydrogel layer along the width direction (*W*_0_) than the length direction (*L*_0_) due to the strong edge effects, leading to initial bending along the width direction (Movie S4). Moreover, the initial bending of the hydrogel film determines the shape-morphing path. Even though a helix shape can be obtained in a lower energy state, the bending along the length direction is hindered due to the difficulty of overcoming the high energy barrier to reverse the short-side rolling. In addition, diagonal rolling, helix, and short-side rolling are still observed as the *L*_0_/*W*_0_ ratio increases from 1 to 12, where the initial length (*L*_0_) of CS hydrogel films increases from 1 mm to 60 mm. The edge effects are further confirmed experimentally by using CS hydrogel films with both *L*_0_ and *W*_0_ close to millimeter size as shown in Figure S4, which increases the probability of short-side rolling. Therefore, various shape-morphing behaviors can be facilely obtained via changing the length-to-width ratios (*L*_0_/*W*_0_) of CS hydrogel films.

To confirm the effect of length-to-width ratios (*L*_0_/*W*_0_) on shape-morphing behaviors, we further explore the relationships between the length-to-width ratios of the swollen CS hydrogel films (*L*_*d*_/*W*_*d*_; *L*_*d*_: the length of the swollen hydrogel film, and *W*_*d*_: the width of the swollen hydrogel film) and various shapes (Figure 3). According to our experimental results (Figure 3), for both diagonal rolling and short-side rolling, the length-to-width ratios (*L*_*d*_/*W*_*d*_) of hydrogel films are close to 1. For the helix, the length-to-width ratios (*L*_*d*_/*W*_*d*_) of hydrogel films are greater than 1. Moreover, the shape evolution of hydrogel films can be indicated via investigating the relationship between the *L*_*d*_/*W*_*d*_ and the *L*_0_/*W*_0_. As shown in Figure 3(c) and Figure S3, it is clear that the *L*_*d*_/*W*_*d*_ ratios (≥1) increase with further increases of *L*_0_/*W*_0_ ratios, indicating that the planar hydrogel films transform into diagonal rolling firstly and then helix mainly. Specially for the hydrogel films with *L*_0_ = 12 mm, the *L*_*d*_/*W*_*d*_ ratios increase with increasing *L*_0_/*W*_0_ ratios firstly and then decrease (Figure 3(c)). Correspondingly, the planar hydrogel film evolves from diagonal rolling, helix, to final short-side rolling. Further decreasing the *W*_0_ (from 5 mm to 1 mm), the shape deformations show differences in the same range of *L*_0_/*W*_0_ ratios from 1 to 12 (Movie S4 and Movie S5). As shown in Figure 3(d) and Figure S4, the *L*_*d*_/*W*_*d*_ (≥1) increases with further increasing *L*_0_/*W*_0_, showing that the planar hydrogel films transform into diagonal rolling firstly, then short-side rolling (increasing probability) and helix mainly. It is worth noting that strong edge effects are particularly significant for the diagonal rolling and short-side rolling (*L*_*d*_/*W*_*d*_: ~1, very small *L*_0_ or/and *W*_0_) [34]. Although we can obtain various shape deformations including diagonal rolling, helix, and short-side rolling via solely changing the length-to-width ratios (Movie S1, Movie S6, and Movie S7), the shape-morphing rules still need to be expounded via studying the geometry effect systematically.

### 2.4. Controllable Shape Morphing via Changing the Thickness

**Figure 4:**
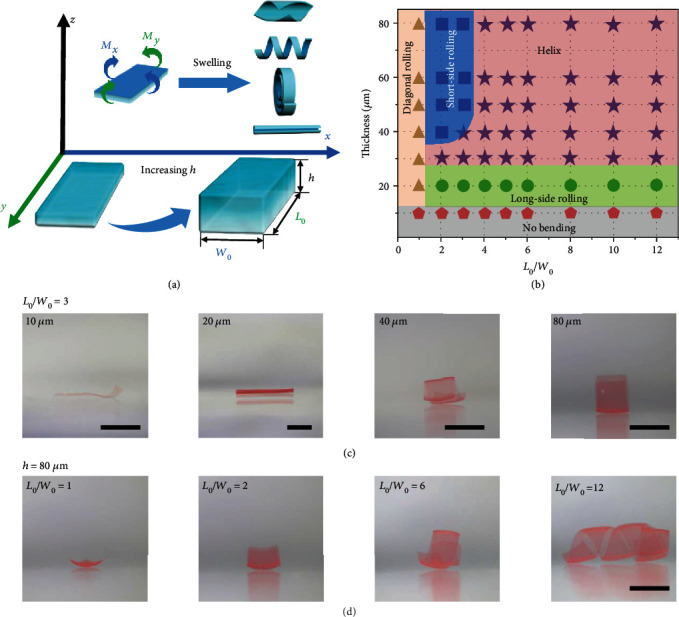
The shape-morphing behaviors of CS hydrogel films via changing the thickness (*h*) and the length-to-width (*L*_0_/*W*_0_) ratios. (a) Schematic illustration of the shape-morphing behaviors (bending moments of **M**_*x*_ at the *x*-axis direction and **M**_*y*_ at the *y*-axis direction) of CS hydrogel films (certain length and width) via changing the thickness (*h*). (b) The phase diagram of shape evolution of CS hydrogel films with different *L*_0_/*W*_0_ ratios and thicknesses (*h*). (c) Pictures of CS hydrogel films with different thicknesses (10 *μ*m, 20 *μ*m, 40 *μ*m, and 80 *μ*m) and certain lengths and widths (*L*_0_/*W*_0_ = 3) show various deformations, including no bending, long-side rolling, helix, and short-side rolling. (d) Pictures of CS hydrogel films with different *L*_0_/*W*_0_ ratios (1, 2, 6, and 12) and certain thickness (80 *μ*m) show various deformations, including diagonal rolling, short-side rolling, and helix. The scale bars are 5 mm.

In order to enhance the shape-morphing editing capability, we further investigate the influences of hydrogel film thickness on shape-morphing behaviors, which is of equal importance for shape-morphing regulation [40, 41]. As discussed previously, for CS hydrogel films with certain initial length and width (*L*_0_: 5 mm, *W*_0_: 5 mm), Young's modulus of both the top and bottom surfaces of the CS hydrogel film decreases with increasing thickness from 20 *μ*m, 40 *μ*m, 60 *μ*m, to 80 *μ*m. Conversely, the decreasing Young's modulus (decreasing cross-linking density) will increase the swelling ratio of the hydrogel film on the one hand, resulting in a larger swelling difference between the top and bottom surfaces of CS hydrogel films. On the other hand, the low Young's modulus of the hydrogel film indicates high deformability according to our previous work [26]. Therefore, increasing the thickness (*h*) of CS hydrogel films would increase the swelling difference (thus increasing Δ*l*) between the top and bottom surfaces of CS hydrogel films, resulting in a large bending moment (**M**_*xy*_) according to Equation (1) that determines the shape morphing of CS hydrogel films (Figure 4(a)). This effect is further experimentally confirmed by employing CS hydrogel films (certain initial length and width: *L*_0_: 9 mm, *W*_0_: 3 mm, *L*_0_/*W*_0_ = 3 : 1) with various thicknesses from 10 *μ*m to 80 *μ*m as shown in Figure S5, which show no bending (10 *μ*m thickness), long-side bending (20 *μ*m thickness), helix (30 *μ*m and 40 *μ*m thicknesses), and short-side bending (50 *μ*m to 80 *μ*m thickness) (Figure 4(b), Movie S8–S11). The shape evolution also agrees with the change of *L*_*d*_/*W*_*d*_ as shown in Figure S5. To reveal the geometry effect (including both *L*_0_/*W*_0_ and *h*) on the shape-morphing behaviors systematically, the effects of the both length-to-width ratio and thickness on the deformation behaviors of CS hydrogel films are described in a phase diagram (Figure 4(b) and Figure S6). Guided by the phase diagram, we can precisely program diverse deformations (i.e., no bending, diagonal rolling, long-side rolling, helix, and short-side rolling) of CS hydrogel films by tuning the thicknesses and/or the length-to-width ratios (Figures 4(c) and 4(d)). Moreover, this robust strategy is not only suitable for CS hydrogel films but also appropriate for sodium alginate (SA) hydrogel films. All shape-morphing behaviors (diagonal rolling, short-side rolling, helix, and long-side rolling) can be facilely achieved by tuning the gradient cross-linking density and geometries of SA hydrogel films (Figure S7), which provides a universal and efficient strategy to precisely control the shape-morphing behaviors of various hydrogels.

### 2.5. Versatile Cooperative Shape Morphing and Actuation

**Figure 5:**
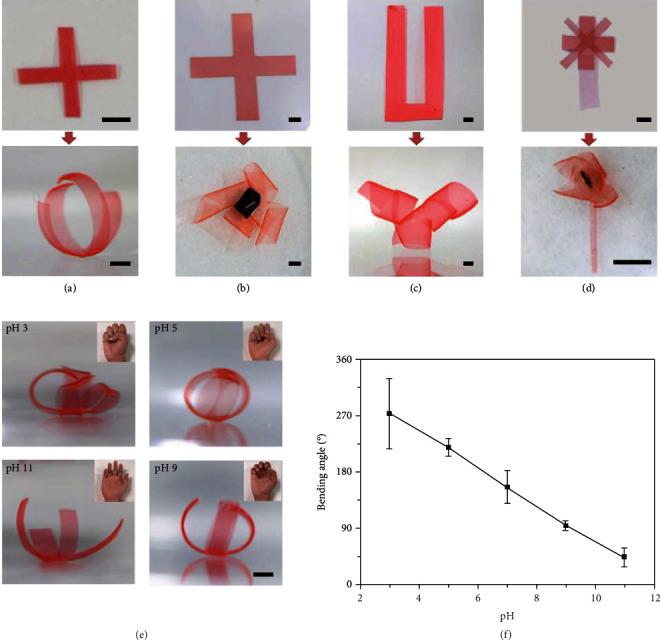
Various types of cooperative shape morphing and actuation. (a–d) Artificial claw (*L*_0_ = 6 mm, *W*_0_ = 1 mm, *h* = 80 *μ*m), artificial windmill (*L*_0_ = 12 mm, *W*_0_ = 5 mm, *h* = 80 *μ*m), an artificial horn (*L*_0_ = 24 mm, *W*_0_ = 12 mm, *h* = 80 *μ*m; length = 20 mm, width = 4 mm), and artificial flower (*L*_0_ = 7 mm, *W*_0_ = 1 mm, *h* = 80 *μ*m; *L*_0_ = 8 mm, *W*_0_ = 3 mm, *h* = 80 *μ*m; *L*_0_ = 12 mm, *W*_0_ = 3 mm, *h* = 20 *μ*m) are customized via modulating the geometries of CS hydrogel films. (e) Actuation of artificial CS hydrogel claws in aqueous solutions with different pH values (pH 3, pH 5, pH 9, and pH 11). The scale bars are 2.5 mm. (f) The bending angles CS hydrogel films decrease as increasing pH due to the protonation/deprotonation of CS chains.

Combining both length-to-width- and thickness-determining effects on programmable shape morphing of CS hydrogel films, we further prepare some complex actuators from planar CS hydrogel films assisted by the art of “kirigami” (Figures 5(a)–5(d)). For cross CS hydrogel films with certain thicknesses (80 *μ*m) but different length-to-width ratios, they quickly change their planar shapes into an artificial claw (Figure 5(a), *L*_0_ = 6 mm, *W*_0_ = 1 mm) and an artificial windmill (Figure 5(b), *L*_0_ = 12 mm, *W*_0_ = 5 mm) after immersing in water for 3 min. Furthermore, an artificial horn can also be obtained upon swelling of a U-shaped hydrogel film (Figure 5(c)), where the feet and bridge of the U-shaped hydrogel film are deformed into helix and short-side rolling, respectively, due to the different length-to-width ratios. In addition, through controlling the thicknesses and the length-to-width ratios of CS hydrogel films precisely, an artificial flower with various parts undergoing distinct deformation modes (i.e., long-side rolling, short-side rolling, and helix) can be facilely achieved (Figure 5(d)).

Besides, owing to the existence of massive cations in CS, the hydrogel films also exhibit pH responsiveness [44]. The deformation of CS hydrogel films can therefore be adjusted by different pH values. Compared with shape morphing in deionized water (pH 7), the swelling degrees of CS hydrogel films are enhanced in an acid condition (pH 3) and reduced in an alkaline condition (pH 11) due to the protonation (increased swelling) and deprotonation (shrinking) of CS chains, respectively (Movie S12 and Movie S13). As shown in Figure S8(a), CS hydrogel films show only two types of shape morphing (diagonal rolling and helix) due to the sharply increased swelling of CS hydrogel films in an aqueous solution at pH 3. On the contrary, the shape morphing of CS hydrogel films changes little in an aqueous solution at pH 11 solution, because CS hydrogel films show slight shrinking (Figure S8(b)). Based on this, we realize dynamic transformations of the underwater artificial claw (Figure 5(e), *h* = 80 *μ*m and *L*_0_/*W*_0_ = 6) by tuning the pH values of environments. The opening/closing of the artificial claw can be switched by dynamically controlling the bending angles of the pH-responsive CS hydrogel fingers. And the bending degrees of CS hydrogel films are quantitively described by the curve of bending angles with respect to pH values (Figure 5(f)). These pH-responsive programmable deformation behaviors of CS hydrogel films make them promising candidates for soft actuators and drug delivery.

## 3. Discussion

In summary, we demonstrate an unprecedented strategy to edit the shape morphing of monocomponent natural polysaccharide hydrogel films via integrating the gradient cross-linking density and geometry effect. Distinct from previous approaches that depend on diverse components or complex manufacturing techniques (photomask-assisted fabrication or 3D printing), the cooperative effect enriches our capabilities to edit the shape morphing of CS hydrogel films solely by tuning their geometries (length-to-width ratios or thicknesses). Moreover, various simple (helix, short-side rolling, and long-side rolling) and complex 3D (artificial claw, horn, and flower) deformations can be easily customized, of which shapes can be further altered by pH. It can be envisioned that this universal strategy can be extended for guiding the design of shape-morphing polymers (for example, hydrogels, liquid crystals, and shape memory polymers) in a simple manner, which also opens new avenues for employing shape-editing polymers for reconfigurable biodevices, soft robotics, and beyond [45–50].

## 4. Materials and Methods

### 4.1. Materials

Chitosan with low average molecular weight, glutaraldehyde aqueous solution (GA, 25.0 wt%), and sodium alginate (SA) were purchased from Sigma-Aldrich. Acetic acid, sodium bicarbonate, and calcium chloride were obtained from Sinopharm Chemical Reagent Co., Ltd. Ammonia solution (NH_3_·H_2_O, 25.0~28.0 wt%) was purchased from Dongjiang Chemical Reagent Co., Ltd. Ultrapure water (≥18 M*Ω*) was used in the experiments. Home-made devices for preparing hydrogel films with certain volumes were made of glass slides containing Teflon spacers with specific sizes (40 mm × 40 mm × 1.5 mm; 80 mm × 80 mm × 2.0 mm).

### 4.2. Fabrication of CS Pregel Solution

CS powders were dissolved in an AA solution (2.0 wt%) and stirred overnight to prepare a CS pregel solution (2.2 wt%). Congo red (0.1 wt%) was added in the solution for staining purpose.

### 4.3. Fabrication of CS Hydrogel Films

A 6.2 mL CS pregel solution (2.2 wt%) was poured into a home-made device (40 mm × 40 mm × 1.5 mm). Then, the GA solution was dropped onto the top surface of the CS pregel solution, which was placed at room temperature for 24 h to form a dried CS hydrogel film. To tune the cross-linking degrees of CS hydrogel films, various concentrations of GA (GA/CS (mass ratio%): 9.28%, 4.64%, 2.32%, 1.16%, 0.58%, and 0.29%) were used for chemical cross-linking of CS. To prepare CS hydrogel films with different thicknesses (10 *μ*m, 20 *μ*m, 30 *μ*m, 40 *μ*m, 50 *μ*m, 60 *μ*m, and 80 *μ*m), various volumes of CS pregel solutions (0.78 mL, 1.55 mL, 2.33 mL, 3.10 mL, 3.88 mL, 4.65 mL, and 6.20 mL) were employed and further cross-linked with GA solutions (0.29%). The obtained dried hydrogel films were cut into strips with designated length-to-width ratios. All dried CS films were firstly swollen in deionized water for 10 min. Subsequently, the deformed hydrogel films were immersed into a 0.01 M NaHCO_3_ solution for 30 min to remove the residual acetic acid. Finally, the hydrogel films were soaked in deionized water for 24 h to reach swelling equilibrium for further use. CS films with large sizes were prepared as mentioned above via a large home-made device (Teflon spacer size: 80 mm × 80 mm × 2.0 mm).

### 4.4. Fabrication of SA Hydrogel Films

1.0 mL and 2.5 mL SA solutions (4.8 wt%) were poured into a home-made device (40 mm × 40 mm × 1.5 mm) and then evaporated at room temperature for 24 h to obtain dried films with certain thicknesses of 20 *μ*m and 50 *μ*m, respectively. Afterwards, the SA hydrogel films were cross-linked by immersing in a 0.05 M calcium chloride (CaCl_2_) solution for 1 min and then dried for 6 h [26]. The dried SA hydrogel films were cut into strips with certain length-width ratios, which were finally immersed into water for further use.

### 4.5. Tunable Deformation of CS Hydrogels in Acid and Alkaline Solutions

To study the shape-morphing differences of hydrogel sheets in acid or alkaline solutions, the dried CS hydrogels were cut into various strips with the same initial width (*W*_0_: 3 mm), different lengths (*L*_0_: 3 mm, 6 mm, 9 mm, 12 mm, 15 mm, 18 mm, 24 mm, 30 mm, and 36 mm), and different thicknesses (*h*: 10 *μ*m, 20 *μ*m, 30 *μ*m, 40 *μ*m, 50 *μ*m, 60 *μ*m, and 80 *μ*m). After removal of the residual acetic acid, the hydrogels were immersed in deionized water for 24 hours to reach the swelling equilibrium. Subsequently, the swollen hydrogels were transferred into the acid solution with pH 3 or the alkaline solution with pH 11 for 24 h to reach the swelling equilibrium. The acid solution with pH 3 was obtained by adding 3.30 mL acetic acid into 1000 mL deionized water, and the alkaline solution with pH 11 was obtained by adding 7.74 mL ammonia hydroxide solution into 1000 mL deionized water.

### 4.6. Characterization

The thicknesses of the dried CS and SA hydrogels were measured under an optical microscope (Nikon Ni-U, Japan). The morphologies of the cross-sectional CS hydrogel films were characterized by a field-emission-scanning electron microscope (FE-SEM, ZEISS SIGMA 300). The shape-morphing behaviors of CS and SA hydrogel films were recorded by a digital camera (Canon, 7D Mark II). The local elasticities of CS hydrogel films were probed with a commercial atomic force microscope (AFM, Dimension Icon, Bruker) in the force volume (FV) mechanical imaging mode [26, 28, 51]. Average elasticity was obtained by measuring five different areas of CS hydrogel films.

## Data Availability

The data that support the findings of this study are available from the paper and/or Supplementary Materials. Additional data related to this paper may be requested from the authors.

## Supplementary Materials

Supplementary 1 Figure S1: the bending angles of CS hydrogel films (width: 3 mm, length: 21 mm, thickness: 80 *μ*m) cross-linked with different concentrations of GA. The scale bars are 10 mm. Figure S2: the curve of the swelling ratio of CS hydrogel films with swelling time after removal of residual acetic acid by a 0.01 M NaHCO_3_ solution. The size of the CS hydrogel film is 5 mm × 5 mm × 80 *μ*m. Figure S3: the digital photos of the shape-morphing behaviors of CS hydrogel films (certain thickness: 80 *μ*m) with different *L*_0_ (36 mm, 24 mm, and 12 mm) in the same range of *L*_0_/*W*_0_ ratios (from 1 to 12). *L*_0_ and *W*_0_ are the initial length and width of the dried CS hydrogel film, respectively. *L*_*d*_ and *W*_*d*_ are the length and width of the swollen hydrogel, respectively. The scale bar is 10 mm. Figure S4: the digital photos of the shape-morphing behaviors of CS hydrogel films (80 *μ*m thickness) with different *W*_0_ (1 mm, 2 mm, 3 mm, 4 mm, and 5 mm) in the same range of *L*_0_/*W*_0_ ratios (from 1 to 12). *L*_0_ and *W*_0_ represent the initial length and width of the dried CS hydrogel film, respectively. *L*_*d*_ and *W*_*d*_ represent the length and width of the swollen hydrogel, respectively. The scale bars are 10 mm. Figure S5: the shape morphing of CS hydrogel films (certain length and width: *L*_0_ = 9 mm, *W*_0_ = 3 mm) changes as the thickness of CS hydrogels increases, resulting in swelling differences (as shown in the inserted pictures). The scale bars are 10 mm. Figure S6: the shape-morphing behaviors of various CS hydrogel films with different *L*_0_/*W*_0_ ratios (from 1, 2, 3, 4, 5, 6, 8, 10, to 12) and thicknesses (from 10 *μ*m, 20 *μ*m, 30 *μ*m, 40 *μ*m, 50 *μ*m, 60 *μ*m, to 80 *μ*m) in the phase diagram. The scale bar is 10 mm. Figure S7: the controllable shape morphing of sodium alginate (SA) hydrogel films via tuning the geometries. (a) Diagonal rolling (10 mm × 10 mm × 50 *μ*m). (b) Short-side rolling (1 mm × 30 mm × 50 *μ*m). (c) Helix (5 mm × 30 mm × 50 *μ*m). (d) Long-side rolling (5 mm × 30 mm × 20 *μ*m). The scale bar is 5 mm. Figure S8: the phase diagrams of the swollen CS hydrogel films with the same width (*W*_0_: 3 mm.), different *L*_0_/*W*_0_ ratios (from 1, 2, 3, 4, 5, 6, 8, 10, to 12), and thicknesses (from 10 *μ*m, 20 *μ*m, 30 *μ*m, 40 *μ*m, 50 *μ*m, 60 *μ*m, to 80 *μ*m) after immersing in acid (pH 3) and alkaline solutions (pH 11). Table S1: Young's modulus of the top and bottom surfaces of CS hydrogel films with different initial thicknesses (20 *μ*m, 40 *μ*m, 60 *μ*m, and 80 *μ*m) after immersing in water for 10 min, then in 0.01 M NaHCO_3_ solution for 30 min, and finally in water for 24 h.

Supplementary 2 Movie S1: the diagonal rolling of CS hydrogel film (12 mm × 12 mm × 80 *μ*m).

Supplementary 3 Movie S2: the helical deformation of CS hydrogel film (36 mm × 6 mm × 80 *μ*m).

Supplementary 4 Movie S3: the helical deformation of CS hydrogel film (36 mm × 9 mm × 80 *μ*m).

Supplementary 5 Movie S4: the short-side rolling of CS hydrogel film (12 mm × 1 mm × 80 *μ*m).

Supplementary 6 Movie S5: the helical deformation of CS hydrogel film (60 mm × 5 mm × 80 *μ*m).

Supplementary 7 Movie S6: the helical deformation of CS hydrogel film (24 mm × 3 mm × 80 *μ*m).

Supplementary 8 Movie S7: the short-side rolling of CS hydrogel film (70 mm × 1 mm × 80 *μ*m).

Supplementary 9 Movie S8: no bending of CS hydrogel film (9 mm × 3 mm × 10 *μ*m).

Supplementary 10 Movie S9: the long-side rolling of CS hydrogel film (9 mm × 3 mm × 20 *μ*m).

Supplementary 11 Movie S10: the helical deformation of CS hydrogel film (9 mm × 3 mm × 30 *μ*m).

Supplementary 12 Movie S11: the short-side rolling of CS hydrogel film (9 mm × 3 mm × 60 *μ*m).

Supplementary 13 Movie S12: swelling of CS hydrogel film (9 mm × 3 mm × 20 *μ*m) in an acid solution (pH 3).

Supplementary 14 Movie S13: shrinking of CS hydrogel film (9 mm × 3 mm × 50 *μ*m) in an alkaline solution (pH 11).
