# Supplementary material for: Editing the Shape Morphing of Monocomponent Natural Polysaccharide Hydrogel Films
Source: Research (Wash D C). 2021 Jun 2;2021:9786128. doi: 10.34133/2021/9786128 (PMC8214511; doi:10.34133/2021/9786128)
Supplement: Supplementary 1 — Figure S1: the bending angles of CS hydrogel films (width: 3 mm, length: 21 mm, thickness: 80 μm) cross-linked with different concentrations of GA. The scale bars are 10 mm. Figure S2: the curve of the swelling ratio of CS hydrogel films with swelling time after removal of residual acetic acid by a 0.01 M NaHCO3 solution. The size of the CS hydrogel film is 5 mm × 5 mm × 80 μm. Figure S3: the digital photos of the shape-morphing behaviors of CS hydrogel films (certain thickness: 80 μm) with different L0 (36 mm, 24 mm, and 12 mm) in the same range of L0/W0 ratios (from 1 to 12). L0 and W0 are the initial length and width of the dried CS hydrogel film, respectively. Ld and Wd are the length and width of the swollen hydrogel, respectively. The scale bar is 10 mm. Figure S4: the digital photos of the shape-morphing behaviors of CS hydrogel films (80 μm thickness) with different W0 (1 mm, 2 mm, 3 mm, 4 mm, and 5 mm) in the same range of L0/W0 ratios (from 1 to 12). L0 and W0 represent the initial length and width of the dried CS hydrogel film, respectively. Ld and Wd represent the length and width of the swollen hydrogel, respectively. The scale bars are 10 mm. Figure S5: the shape morphing of CS hydrogel films (certain length and width: L0 = 9 mm, W0 = 3 mm) changes as the thickness of CS hydrogels increases, resulting in swelling differences (as shown in the inserted pictures). The scale bars are 10 mm. Figure S6: the shape-morphing behaviors of various CS hydrogel films with different L0/W0 ratios (from 1, 2, 3, 4, 5, 6, 8, 10, to 12) and thicknesses (from 10 μm, 20 μm, 30 μm, 40 μm, 50 μm, 60 μm, to 80 μm) in the phase diagram. The scale bar is 10 mm. Figure S7: the controllable shape morphing of sodium alginate (SA) hydrogel films via tuning the geometries. (a) Diagonal rolling (10 mm × 10 mm × 50 μm). (b) Short-side rolling (1 mm × 30 mm × 50 μm). (c) Helix (5 mm × 30 mm × 50 μm). (d) Long-side rolling (5 mm × 30 mm × 20 μm). The scale bar is 5 mm. Figure S8: the p [file 9786128.f1.docx]

**Editing the Shape Morphing of Mono-component Natural Polysaccharide Hydrogel Films**

Hao Hu,^1,3†^ Chao Huang,^1†^ Massimiliano Galluzzi,^2^ Qiang Ye,^3^* Rui Xiao,^4^ Xuefeng Yu,^2^ and Xuemin Du^1^*

*^1^Institute of Biomedical & Health Engineering, Shenzhen Institute of Advanced Technology (SIAT), Chinese Academy of Sciences (CAS), Shenzhen, 518035, China.*

*^2^Institute of Advanced Materials Science and Engineering, Shenzhen Institute of Advanced Technology (SIAT), Chinese Academy of Sciences (CAS), Shenzhen, 518035, China.*

*^3^Key Laboratory of Polymeric Materials and Application Technology of Hunan Province, Key Laboratory of Environmentally Friendly Chemistry and Applications of Ministry of Education, School of Chemistry, Xiangtan University, Xiangtan 411105, China.*

*^4^ State Key Laboratory of Fluid Power & Mechatronic System, Key Laboratory of Soft Machines and Smart Devices of Zhejiang Province, Department of Engineering Mechanics, Zhejiang University, Hangzhou, 310027, China.*

^†^These authors contributed equally to this work.

Correspondence should be addressed to X. Du; [xm.du@siat.ac.cn](mailto:xm.du@siat.ac.cn), Q. Ye; [qiang_ye@xtu.edu.cn](mailto:qiang_ye@xtu.edu.cn)

**Supplementary Materials.**

**Figure S1**: The bending angles of CS hydrogel films (width: 3 mm, length: 21 mm, thickness: 80 μm) crosslinked with different concentrations of GA. The scale bars are 10 mm.

**Figure S2**: The curve of swelling ratio of CS hydrogel films with swelling time after removal of residual acetic acid by a 0.01 M NaHCO_3_ solution. The size of CS hydrogel film is 5 mm × 5 mm × 80 μm.

**Figure S3**: The digital photos of the shape-morphing behaviors of CS hydrogel films (certain thickness: 80 μm) with different *L_0_* (36 mm, 24 mm, and 12 mm) in the same range of *L_0_*/*W_0_* ratios (from 1 to 12). *L_0_* and *W_0_* are the initial length and width of dried CS hydrogel film, respectively. *L_d_* and *W_d_* are the length and width of the swollen hydrogel, respectively. The scale bar is 10 mm.

**Figure S4**: The digital photos of the shape-morphing behaviors of CS hydrogel films (80-μm thickness) with different *W_0_* (1 mm, 2 mm, 3 mm, 4 mm, and 5 mm) in the same range of *L_0_*/*W_0_* ratios (from 1 to 12). *L_0_* and *W_0_* represent the initial length and width of dried CS hydrogel film, respectively. *L_d_* and *W_d_* represent the length and width of the swollen hydrogel, respectively. The scale bars are 10 mm.

**Figure S5**: The shape morphing of CS hydrogel films (certain length and width: *L_0_ =* 9 mm, *W_0_ =* 3 mm) changes as increasing the thickness of CS hydrogels, resulting in swelling differences (as shown in the inserted pictures). The scale bars are 10 mm.

**Figure S6**: The shape-morphing behaviors of various CS hydrogel films with different L_0_/W_0_ ratios (from 1, 2, 3, 4, 5, 6, 8, 10, to 12) and thicknesses (from 10 μm, 20 μm, 30 μm, 40 μm, 50 μm, 60 μm, to 80 μm) in the phase diagram. The scale bar is 10 mm.

**Figure S7**: The controllable shape morphing of sodium alginate (SA) hydrogel films via tuning the geometries. (a) Diagonal rolling (10 mm × 10 mm × 50 μm). (b) Short-side rolling (1 mm × 30 mm × 50 μm). (c) Helix (5 mm × 30 mm × 50 μm). (d) Long-side rolling (5 mm × 30 mm × 20 μm). The scale bar is 5 mm.

**Figure S8**: The phase diagrams of the swollen CS hydrogel films with the same width (*W_0_*: 3 mm.), different L_0_/W_0_ ratios (from 1, 2, 3, 4, 5, 6, 8, 10, to 12), and thicknesses (from 10 μm, 20 μm, 30 μm, 40 μm, 50 μm, 60 μm, to 80 μm) after immersing in acid (pH 3) and alkaline solutions (pH 11).

**Table S1**: Young’s modulus of the top and bottom surfaces of CS hydrogel films with different initial thicknesses (20 μm, 40 μm, 60 μm, and 80 μm) after immersing in water for 10 min, then in 0.01 M NaHCO_3_ solution for 30 min, and finally in water for 24 h.


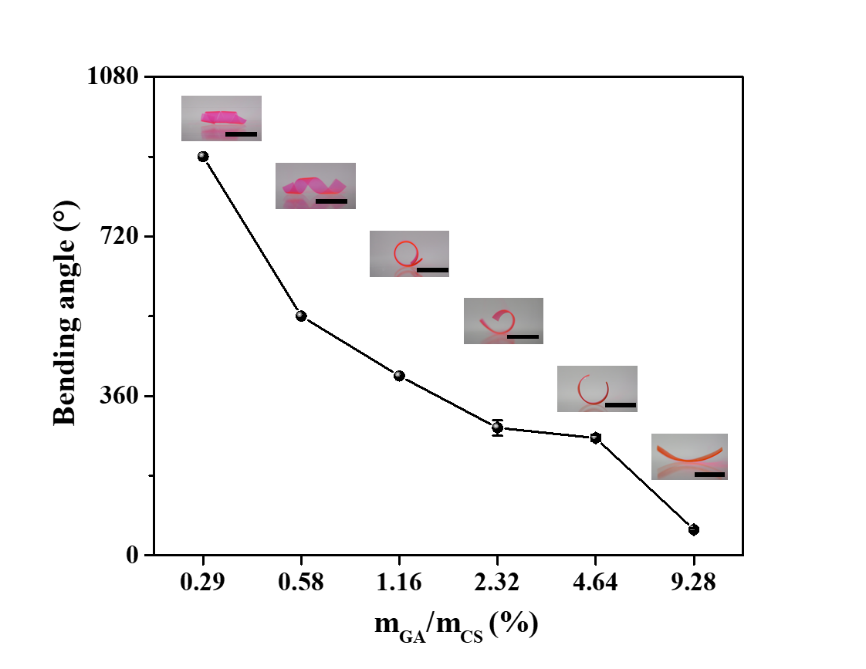


**Figure S1.** The bending angles of CS hydrogel films (width: 3 mm, length: 21 mm, thickness: 80 μm) crosslinked with different concentrations of GA. The scale bars are 10 mm.


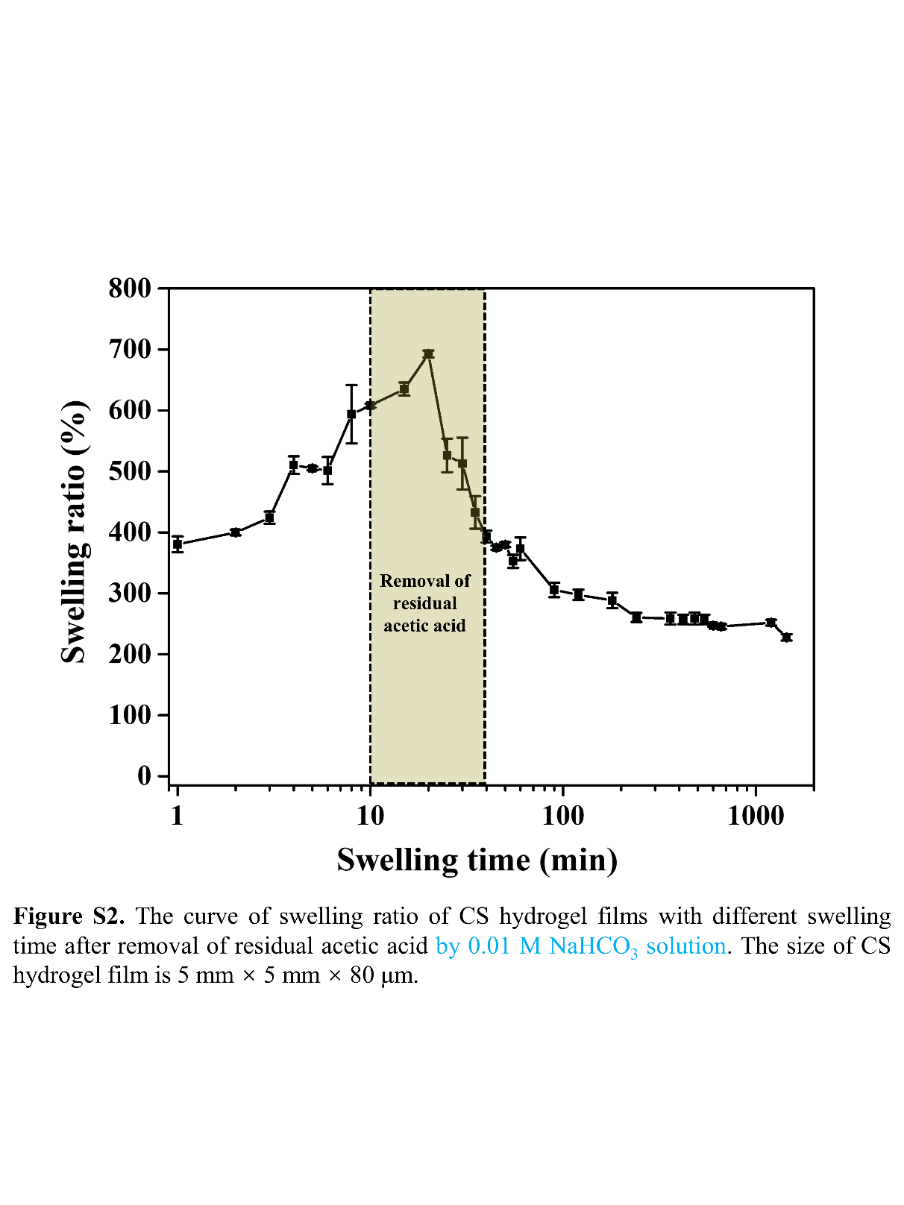
**Figure S2.** The curve of swelling ratio of CS hydrogel films with swelling time after removal of residual acetic acid by a 0.01 M NaHCO_3_ solution. The size of CS hydrogel film is 5 mm × 5 mm × 80 μm.

**Table S1.** Young’s modulus of the top and bottom surfaces of CS hydrogel films with different initial thicknesses (20 μm, 40 μm, 60 μm, and 80 μm) after immersing in water for 10 min, then in 0.01 M NaHCO_3_ solution for 30 min, and finally in water for 24 h.

| **Thickness (μm)** | **Top surface (MPa)** | **Bottom surface (MPa)** | **ΔE (MPa)** |
| --- | --- | --- | --- |
| **20** | 6.41 ± 0.49 | 1.06 ± 0.13 | 5.35 ± 0.49 |
| **40** | 2.40 ± 0.02 | 0.76 ± 0.02 | 1.64 ± 0.02 |
| **60** | 1.43 ± 0.11 | 0.39 ± 0.06 | 1.04 ± 0.11 |
| **80** | 0.94 ± 0.05 | 0.08 ± 0.01 | 0.86 ± 0.05 |


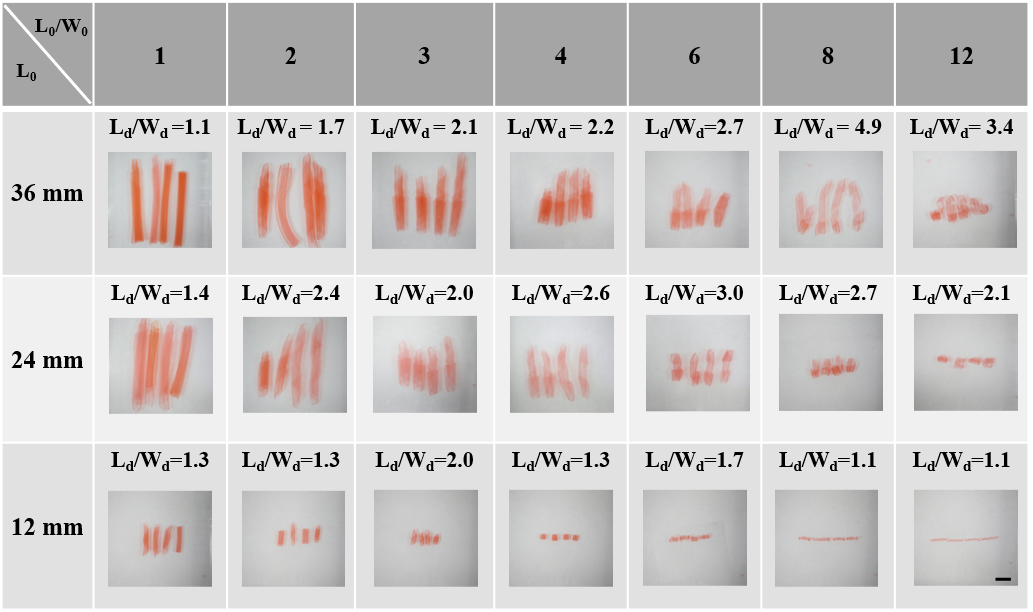


**Figure S3.** The digital photos of the shape-morphing behaviors of CS hydrogel films (certain thickness: 80 μm) with different *L_0_* (36 mm, 24 mm, and 12 mm) in the same range of *L_0_*/*W_0_* ratios (from 1 to 12). *L_0_* and *W_0_* are the initial length and width of dried CS hydrogel film, respectively. *L_d_* and *W_d_* are the length and width of the swollen hydrogel, respectively. The scale bar is 10 mm.

**
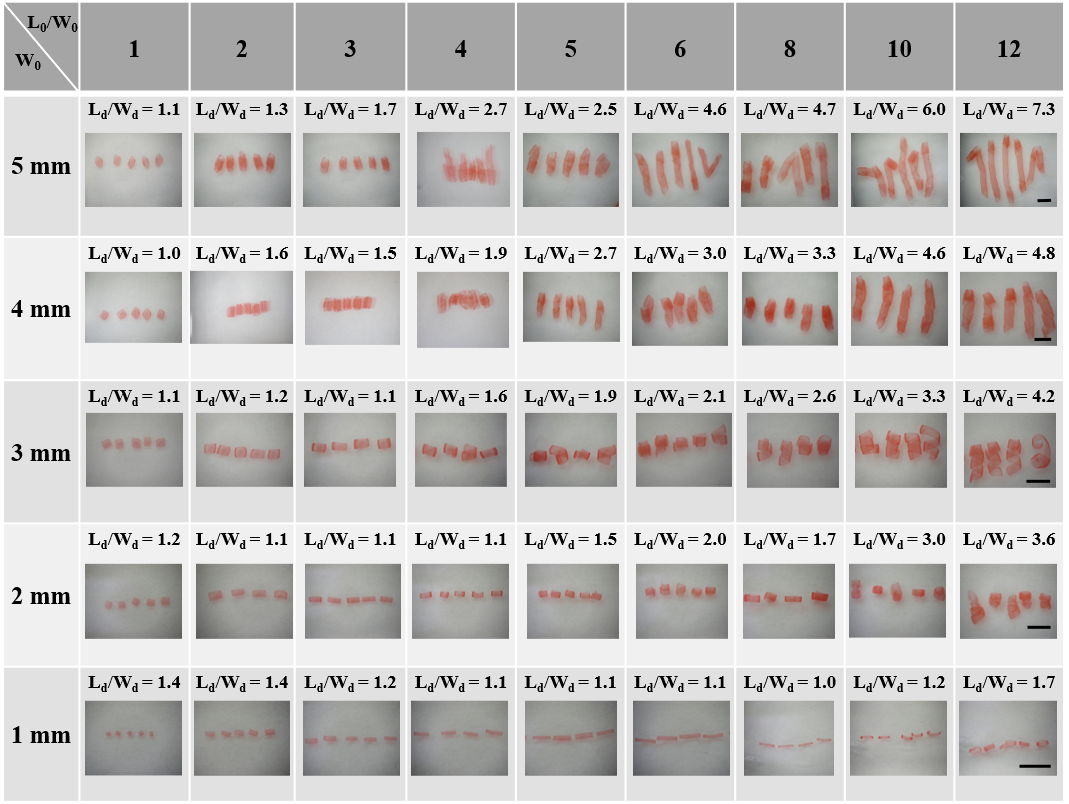
**

**Figure S4.** The digital photos of the shape-morphing behaviors of CS hydrogel films (80-μm thickness) with different *W_0_* (1 mm, 2 mm, 3 mm, 4 mm, and 5 mm) in the same range of *L_0_*/*W_0_* ratios (from 1 to 12). *L_0_* and *W_0_* represent the initial length and width of dried CS hydrogel film, respectively. *L_d_* and *W_d_* represent the length and width of the swollen hydrogel , respectively. The scale bars are 10 mm.


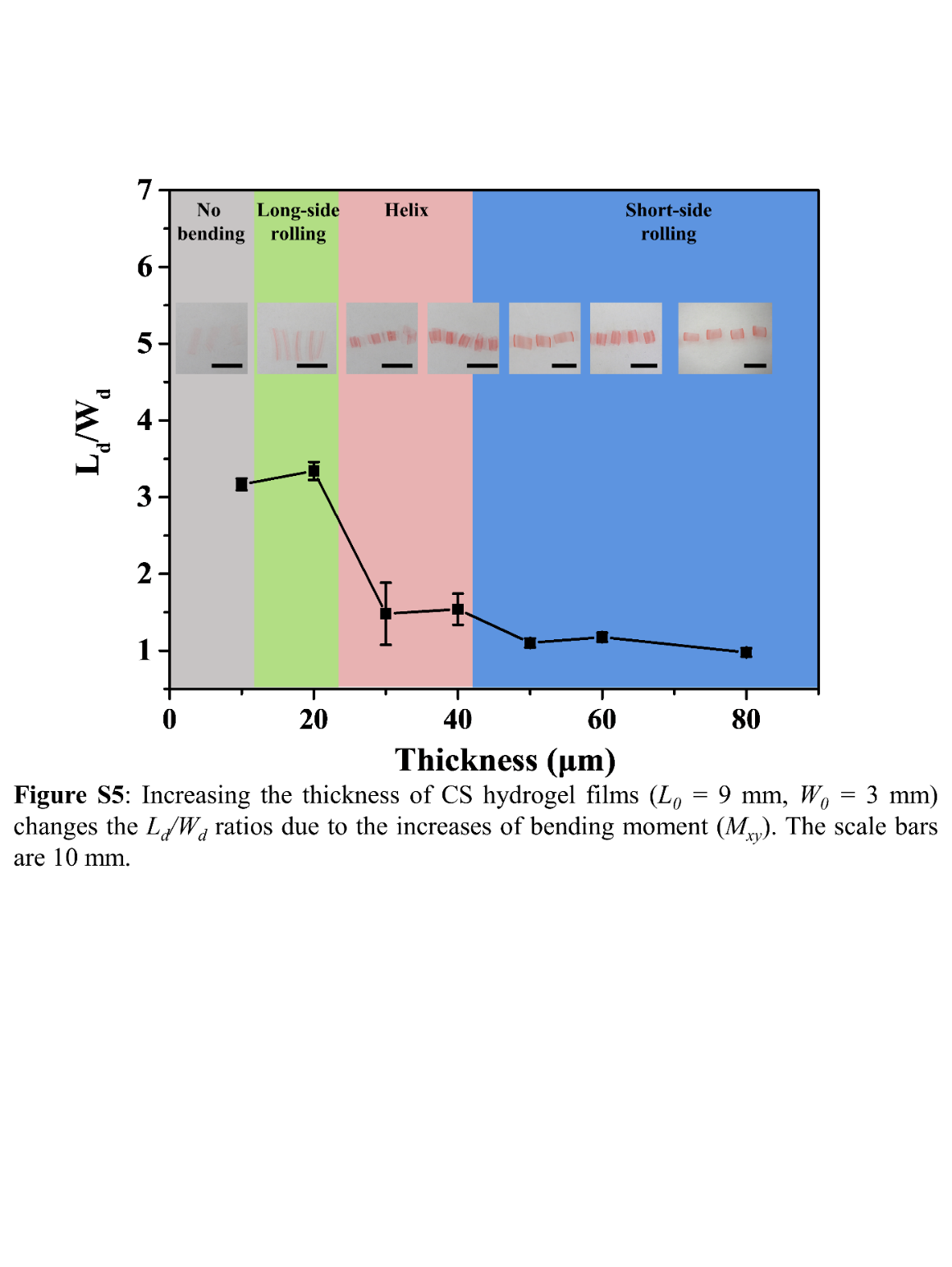
**Figure S5**: The shape morphing of CS hydrogel films (certain length and width: *L_0_ =* 9 mm, *W_0_ =* 3 mm) changes as increasing the thickness of CS hydrogels, resulting in swelling differences (as shown in the inserted pictures). The scale bars are 10 mm.


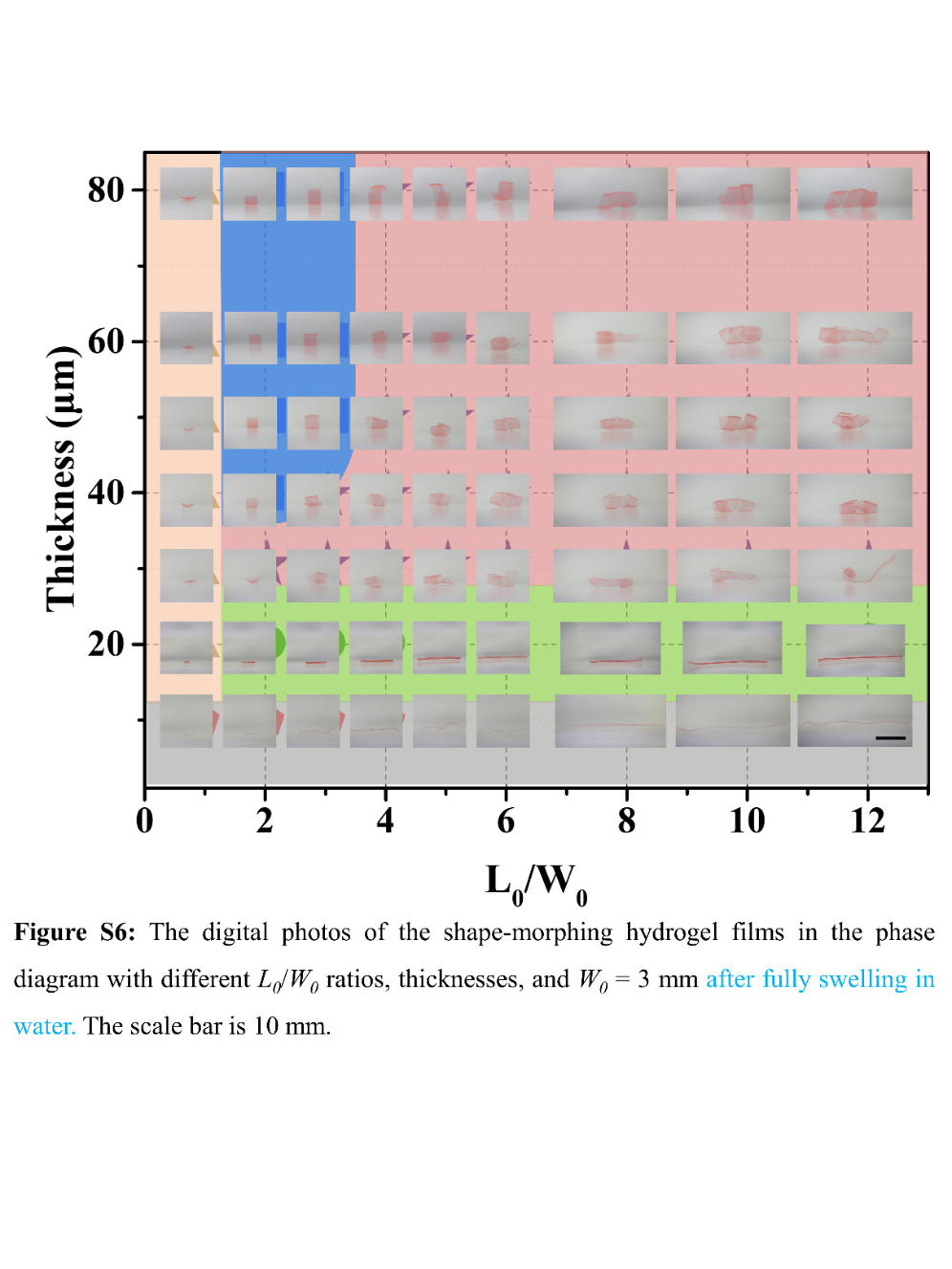


**Figure S6.** The shape-morphing behaviors of various CS hydrogel films with different L_0_/W_0_ ratios (from 1, 2, 3, 4, 5, 6, 8, 10, to 12) and thicknesses (from 10 μm, 20 μm, 30 μm, 40 μm, 50 μm, 60 μm, to 80 μm) in the phase diagram. The scale bar is 10 mm.


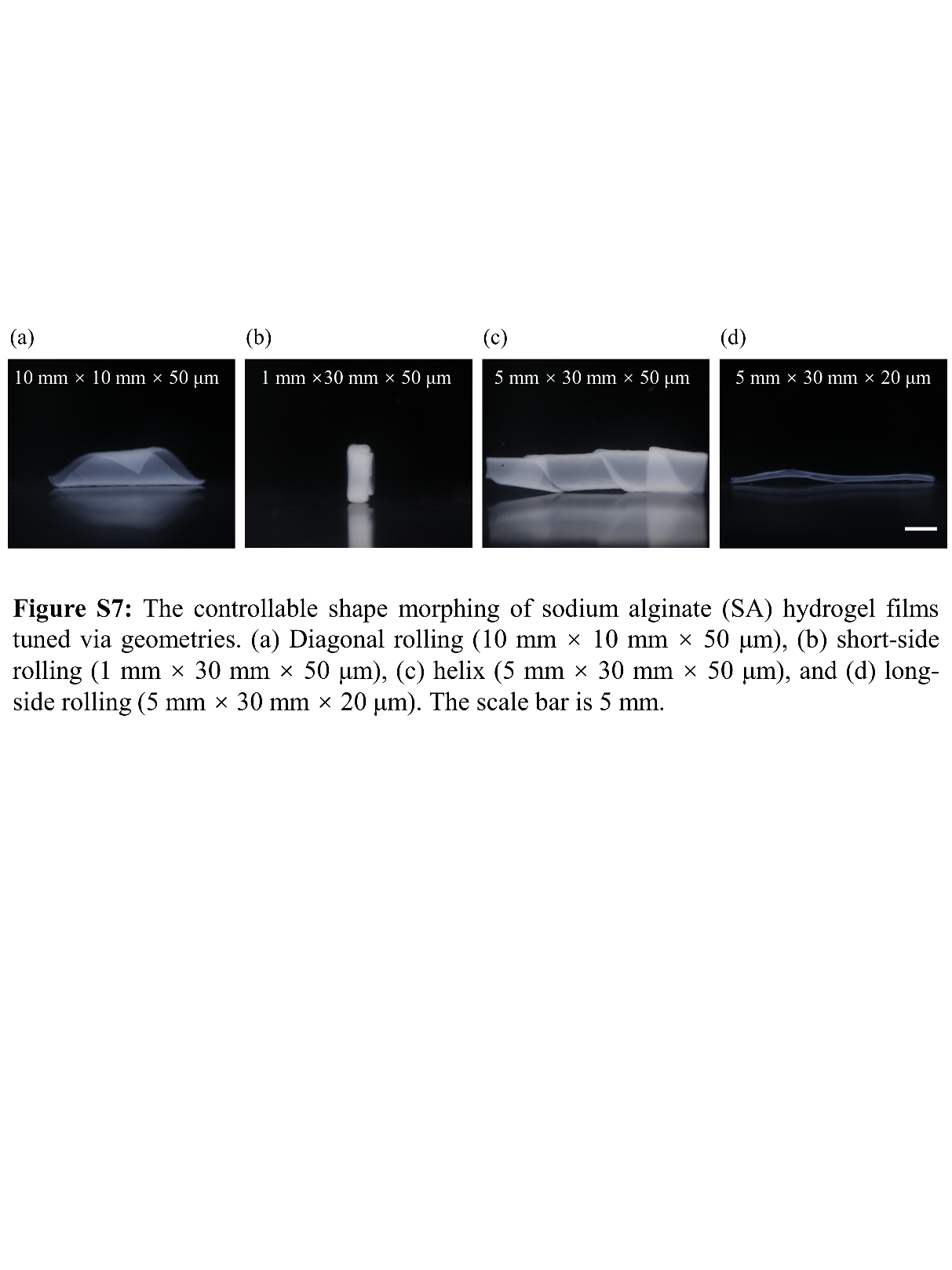


**Figure S7.** The controllable shape morphing of sodium alginate (SA) hydrogel films via tuning the geometries. (a) Diagonal rolling (10 mm × 10 mm × 50 μm). (b) Short-side rolling (1 mm × 30 mm × 50 μm). (c) Helix (5 mm × 30 mm × 50 μm). (d) Long-side rolling (5 mm × 30 mm × 20 μm). The scale bar is 5 mm.


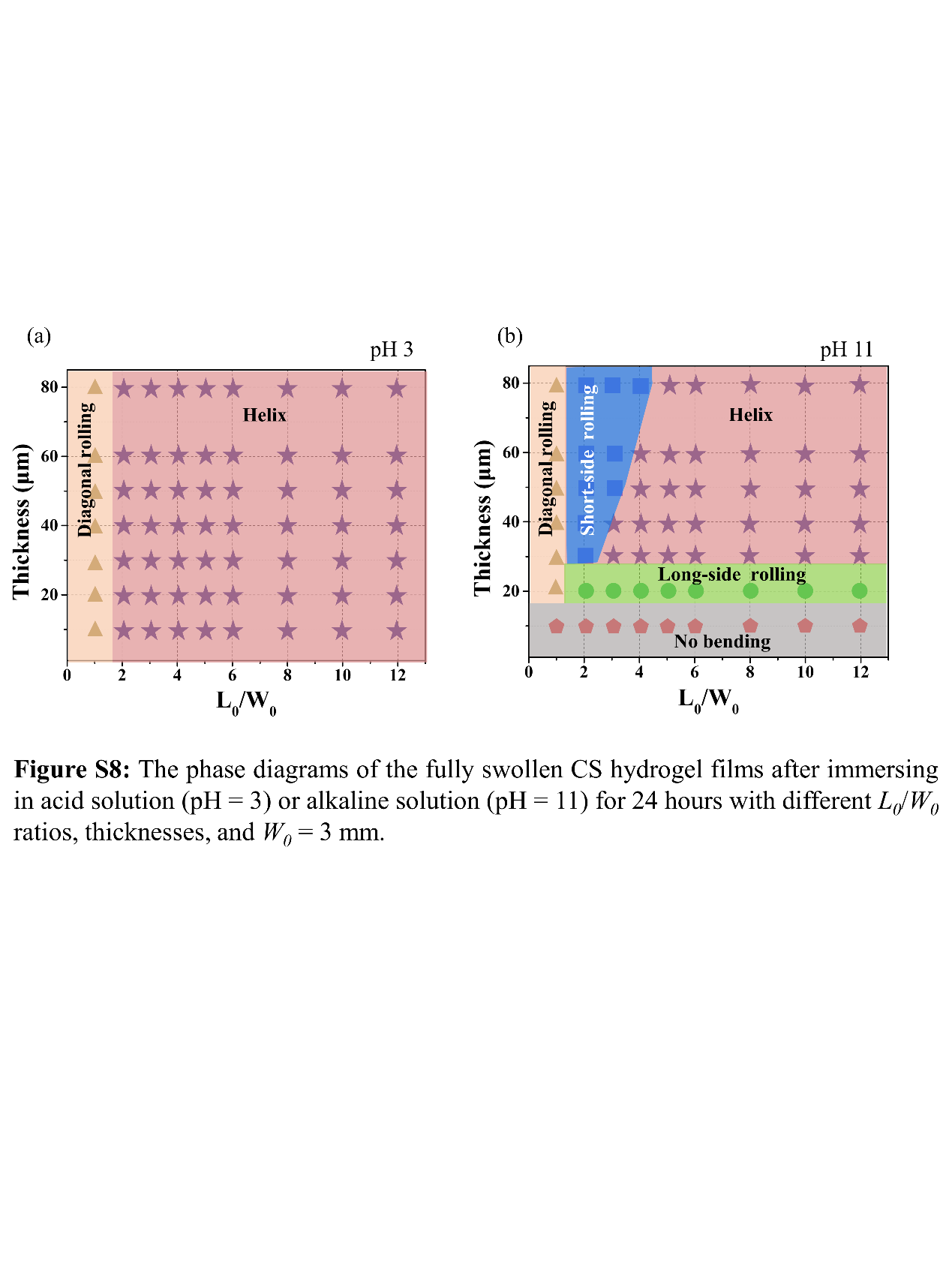


**Figure S8**: The phase diagrams of the swollen CS hydrogel films with the same width (*W_0_*: 3 mm.), different L_0_/W_0_ ratios (from 1, 2, 3, 4, 5, 6, 8, 10, to 12), and thicknesses (from 10 μm, 20 μm, 30 μm, 40 μm, 50 μm, 60 μm, to 80 μm) after immersing in acid (pH 3) and alkaline solutions (pH 11).
